# Supplementary material for: Nitric oxide inhibits ten-eleven translocation DNA demethylases to regulate 5mC and 5hmC across the genome
Source: Nat Commun. 2025 Feb 18;16:1732. doi: 10.1038/s41467-025-56928-1 (PMC11836389; doi:10.1038/s41467-025-56928-1)
Supplement: Supplementary file 1 — Supplementary Information [file 41467_2025_56928_MOESM1_ESM.pdf]

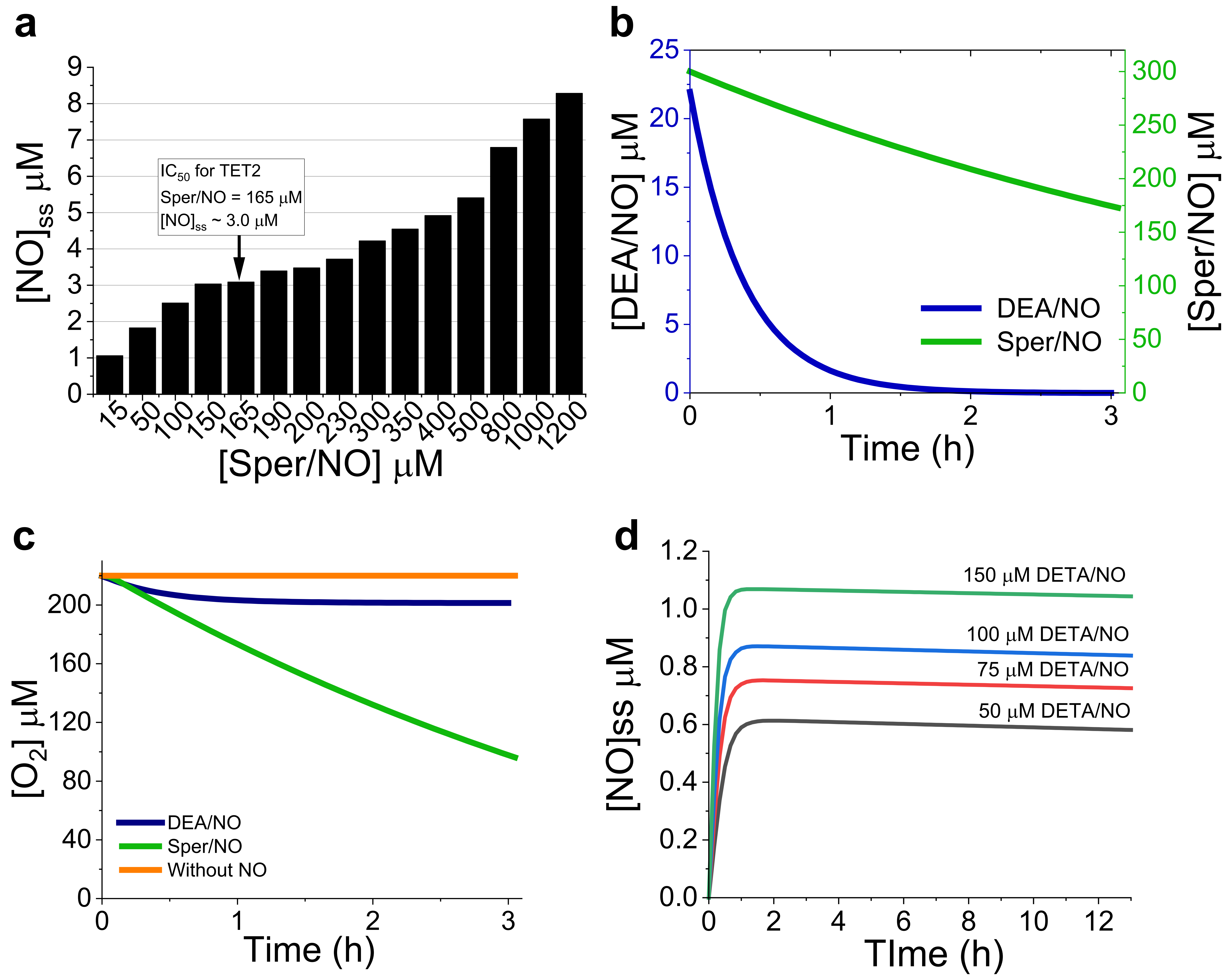

Supplementary Figure 1: Modeling Simulations of NO, O_2, and NO donor concentrations. To simulate the kinetics of the NO donor NO release its subsequent reaction with O_2, we utilized differential equation-solving that involved two main processes: the first-order decay of the NO donor molecules (DEA/NO $t_{1/2}$ = 16 min, Sper/NO $t_{1/2}$ = 230 min., DETA/NO $t_{1/2}$ = 22 hours) and the autooxidation of NO in the presence of O_2 to form nitrogen dioxide (n = 1). **a** Steady-state NO concentrations from the NO donor Sper/NO simulated under the conditions used for experiments in **Figure 1a, b and f**. **b** Simulated first-order decay of DEA/NO and Sper/NO at concentrations used for experiments in **Figure 1d**, (25 μM DEA/NO and 300 μM Sper/NO). **c** Simulated changes in O_2 concentration via the reaction of O_2 with NO in the autooxidation reaction (starting with 25 μM DEA/NO and 300 μM Sper/NO). **d** Steady-state NO concentrations from the NO donor DETA/NO simulated under the conditions used for experiments in **Figure 1h**.

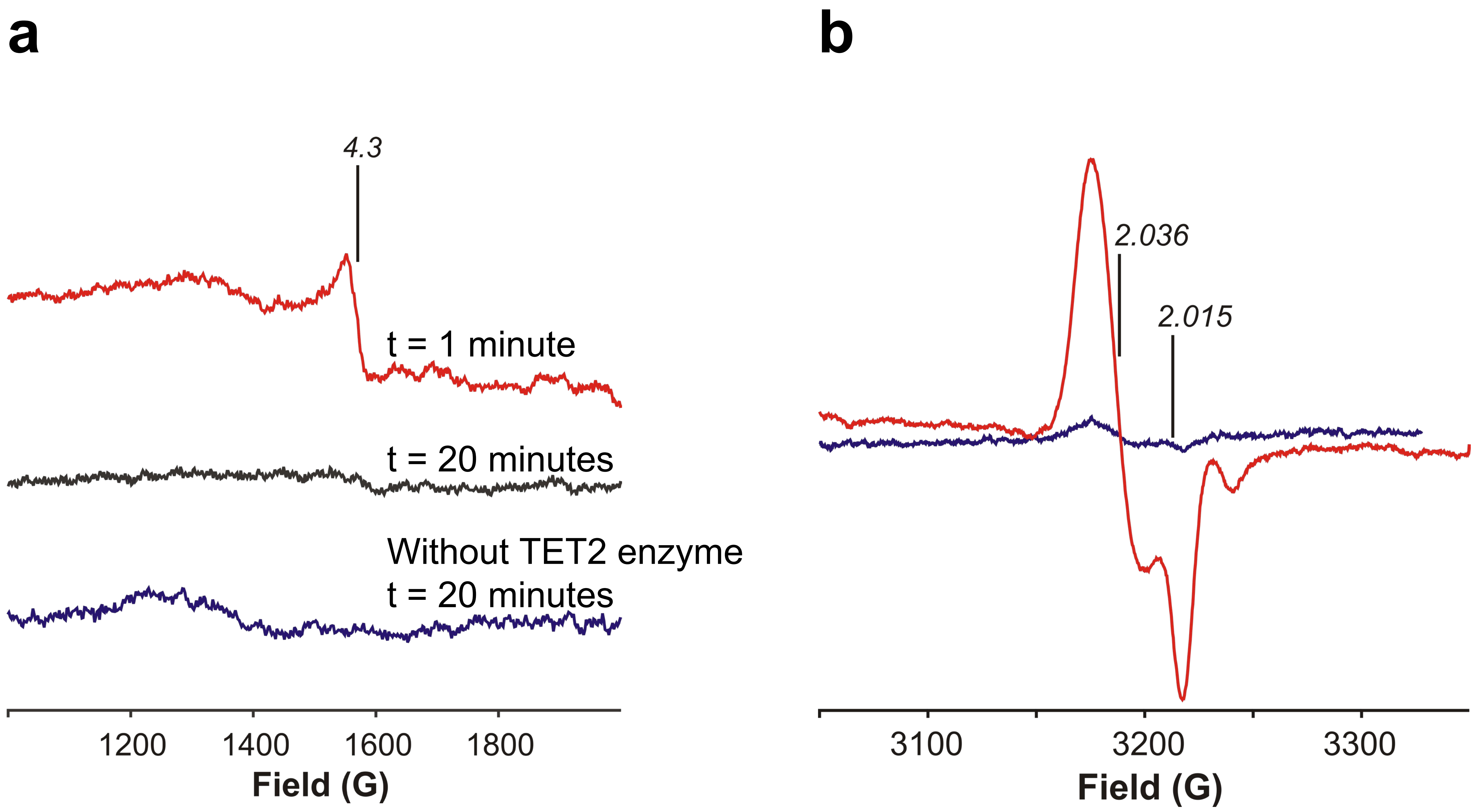

Supplementary Figure 2: The reaction of NO with TET2 does not form a mononitrosyl.
a Representative X-Band (T = 8K) EPR spectra of full length TET2 protein treated with Sper/NO (100 μM) and all substrates and cofactors for 1 min (red line) and 20 min (black line). Control reaction (blue line) is the complete reaction without TET2 after 20 min, *n* = 3. Spectrum is indicative of a non-Heme NO-bound DNIC. **b** 77K EPR spectra of TET2-DNIC protein after NO addition (t= 0, Red) and after NO disappearance (t= > 3 h incubation, blue) demonstrating loss of TET2-DNIC. *n* = 1.

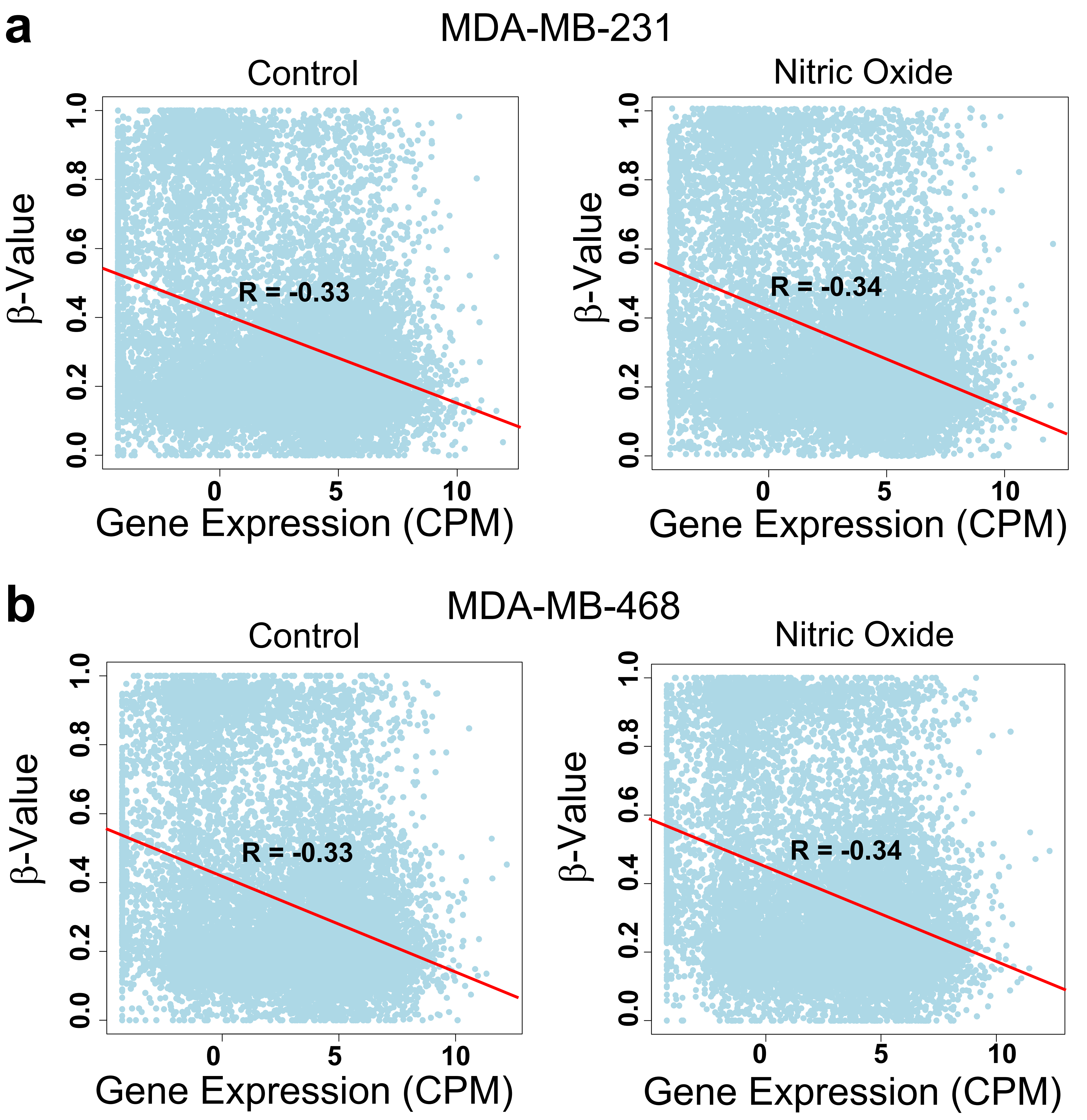

Supplementary Figure 3: NO-mediated increases in 5mC are negatively associated with gene expression in TNBC cells globally. In **a** MDA-MB-231 cells, and **b** MDA-MB-468 cells there was a clear negative correlation between β-value (5mC) and gene expression (expressed as TMM (trimmed mean of M-values) normalized CPM (counts per million) reads) for both control and NO treatment groups. *n* = 2 biological replicates/cell type.

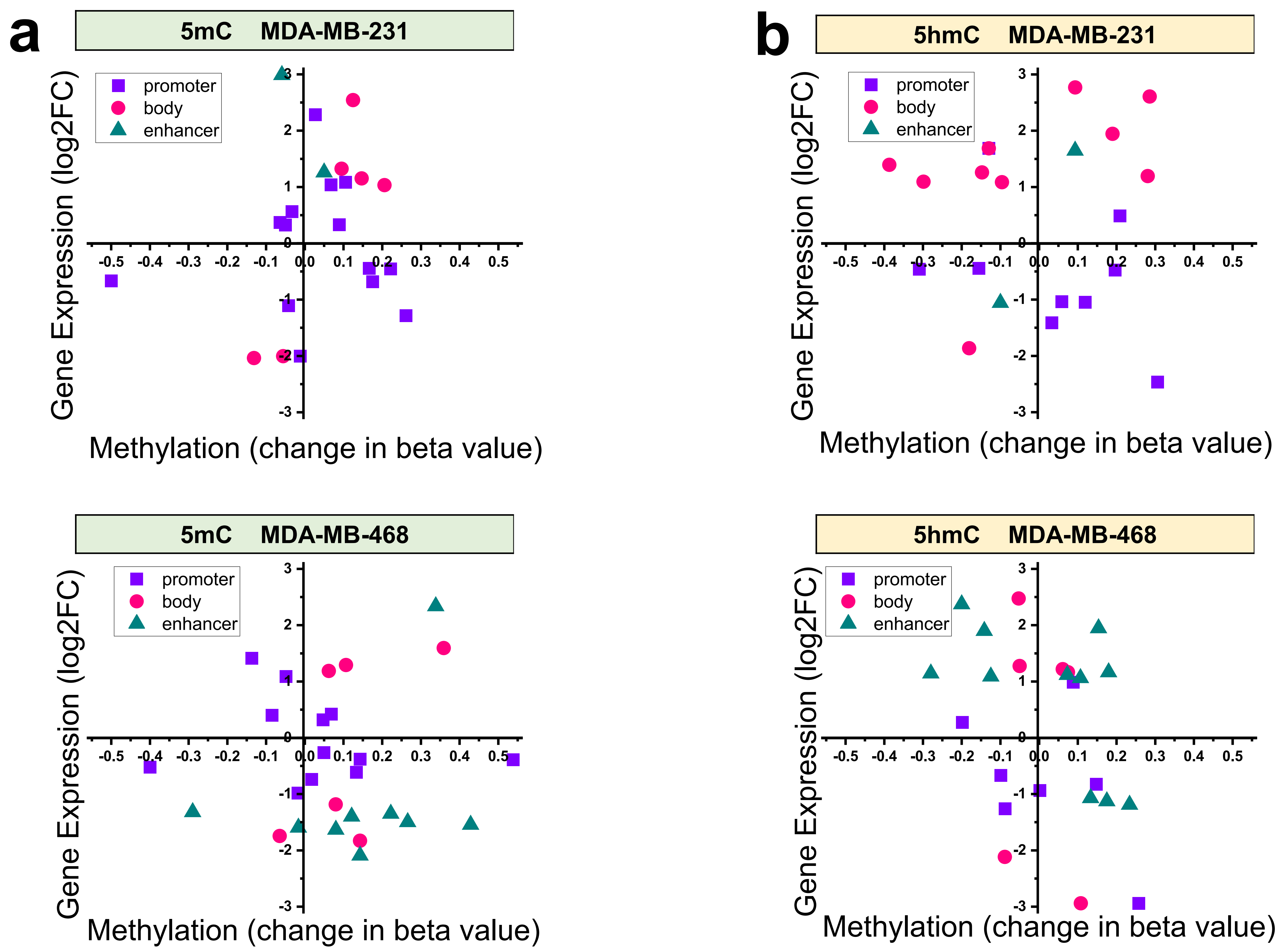

Supplementary Figure 4: Trends in gene expression that are associated with changes in 5mC and 5hmC at promoter, gene body, and enhancer regions. Analysis of oxRRBS and mRNA-seq data from MDA-MB-231 and MDA-MB-468 cells treated with or without DETA/NO (100 µM; 10 days). **a** The relationship between hyper- and hypo-differentially methylated positions (DMP) and changes in associated genes. **b** The relationship between hyper- and hypo-differentially hydroxymethylated positions (DhMP) and changes in associated genes across all annotated sites in both cell types. *P-value* < 0.05, mean difference in abs (beta value) of ≥ 0.1 according to RnBeads. *n* = 2 biological replicates/cell type.

Pathway Annotation	Common Genes	*P-value*	FDR
Tryptophan metabolism	CYP1A1, AOX1, IDO1	2.79E-05	9.87E-04
Jak-STAT signaling pathway	AOX1, IL6, IL6R, IL7R, STAT4	5.49E-05	9.87E-04
African trypanosomiasis	IL6, IDO1	5.04E-04	6.04E-03
Hematopoietic cell lineage	IL6, IL6R, IL7R	7.89E-04	7.10E-03
Pathways in cancer	COL4A6, RASGRP3, IL6, IL6R, IL7R, STAT4, WNT5A	1.45E-03	1.05E-02
Inflammatory bowel disease (IBD)	IL6, STAT4	2.61E-03	1.44E-02
Retinol metabolism	CYP1A1, AOX1	2.84E-03	1.44E-02
RIG-I-like receptor signaling pathway	IFIH1, ISG15	3.22E-03	1.44E-02
PPAR signaling pathway	OLR1, SCD	4.07E-03	1.44E-02
Synaptic vesicle cycle	RIMS1, SLC6A7	4.38E-03	1.44E-02
EGFR tyrosine kinase inhibitor resistance	IL6, IL6R	4.53E-03	1.44E-02
Hepatitis B	IL6, IFIH1, STAT4	4.79E-03	1.44E-02
Influenza A	IL6, IFIH1, RSAD2	5.68E-03	1.57E-02
Protein digestion and absorption	COL4A6, COL17A1	7.58E-03	1.84E-02
Cytokine-cytokine receptor interaction	IL6, IL6R, IL7R, IL1RL1	7.73E-03	1.84E-02
Viral protein interaction with cytokine and cytokine receptor	IL6, IL6R	8.72E-03	1.84E-02
AGE-RAGE signaling pathway in diabetic complications	COL4A6, IL6	8.72E-03	1.84E-02
Amoebiasis	COL4A6, IL6	9.21E-03	1.84E-02
Th17 cell differentiation	IL6, IL6R	1.05E-02	1.94E-02
HIF-1 signaling pathway	IL6, IL6R	1.10E-02	1.94E-02
Rap1 signaling pathway	RAPGEF4, DRD2, RASGRP3	1.18E-02	1.94E-02
Leukocyte transendothelial migration	RAPGEF4, NCF2	1.19E-02	1.94E-02
Human papillomavirus infection	COL4A6, WNT5A, OASL, ISG15	1.24E-02	1.94E-02
PI3K-Akt signaling pathway	COL4A6, IL6, IL6R, IL7R	1.64E-02	2.46E-02
FoxO signaling pathway	IL6, IL7R	1.81E-02	2.60E-02
Measles	IL6, IFIH1	2.07E-02	2.87E-02
Phospholipase D signaling pathway	RAPGEF4, PTGFR	2.49E-02	3.25E-02
Non-alcoholic fatty liver disease (NAFLD)	IL6, IL6R	2.53E-02	3.25E-02
Phagosome	NCF2, OLR1	2.66E-02	3.31E-02
Epstein-Barr virus infection	IL6, ISG15	5.38E-02	6.26E-02
Pathogenic Escherichia coli infection	MYH15, IL6	5.44E-02	6.26E-02
Neuroactive ligand-receptor interaction	GPR83, DRD2, PTGFR	5.56E-02	6.26E-02

Supplementary Table 1: Pathway analysis of NO-regulated genes in TNBC cell lines. Gene Set Enrichment Analysis (GSEA) of the 90 differentially expressed genes that were regulated by NO and common to both MDA-MD-231 and MDA-MB-468 cells ($|log_2FC| > 1$, FDR < 0.05). KEGG pathways with FDR < 0.05 are shown for 231 and 468 cells. Gene set enrichment analysis (GSEA) was completed using OmicPath (https://github.com/CBIIT-CGBB/OmicPath) with hypergeometric testing implemented. P values from all tests were also adjusted to generate FDR (False Discovery Rate) with built-in Benjamini-Hochberg method.

5mC

5mC			NO vs. Control	
**Cell**	**Region**	**Gene**	**Δ β value**	**Δ Gene expression (logFC)**
MDA-MB-231	Promoter	RGS4	0.0679	1.0421
		PLB1	0.0887	0.3288
		GJC2	0.0273	2.2828
		DNAH1	0.1053	1.0877
		TMEM139	0.2611	-1.2832
		AC137932.4	0.1667	-0.4454
		SMAD4	0.2217	-0.4522
		SYTL1	0.1750	-0.6796
		RP1-151F17.2	-0.0359	
		FTSJ3	-0.0641	0.3706
		TMEM140	-0.0333	0.5613
		COG5	-0.0500	0.3271
		AC017074.2	-0.0120	-2.0043
		PLEKHG4	-0.5000	-0.6662
		AC068580.7	-0.0417	-1.1071
	Gene body	GBP4	0.1468	1.1515
		GPR115	0.1247	2.5429
		PLCZ1	0.0952	1.3236
		OVGP1	0.2058	1.0334
		AC017074.2	-0.0557	-2.0043
		FGFBP1	-0.1310	-2.0368
	T.E.	EGFR-AS1	0.0499	1.2581
	S.E.	KLHL38	-0.0592	2.9883
MDA-MB-468	Promoter	AAK1	0.0683	0.4234
		DPP9	0.0473	0.3173
		PRR15L	0.1425	-0.3773
		ARHGEF19	0.5385	-0.3883
		RP11-147L13.13	0.0178	-0.7378
		CTC-559E9.8	0.1333	-0.6127
		AGA	0.0498	-0.2655
		TPRXL	-0.0847	0.4024
		CLIP4	-0.0488	1.0890
		CPA4	-0.1370	1.4113
		TMEM139	-0.4000	-0.5227
		AP001469.5	-0.0181	-0.9807
	Gene body	OLR1	0.1067	1.2925
		ZBED2	0.3591	1.5933
		AC017060.1	0.0621	1.1867
		ADH1C	0.1429	-1.8285
		RP11-401P9.5	0.0801	-1.1844
		RP11-88E10.4	-0.0648	-1.7452
	Typical enhancer	COL15A1	0.3385	2.3375
		SEMA3E	0.2658	-1.4976
		KIT	0.1429	-2.0923
		STAC2	0.4286	-1.5455
		CYP4F8	0.0802	-1.6320
		FAM107A	0.2223	-1.3488
		BACH1-IT2	0.1212	-1.3999
		GALNT12	-0.2896	-1.3202
	S.E.	TACR1	-0.0166	-1.5961

Supplementary Table 2

Supplementary Tables 2: NO-regulated genes and their association with changes in methylation (5mC) at specific genomic loci. β-values (5mC) at gene-regulatory loci (as determined by RnBeads) are paired with the expression changes in their associated genes in MDA-MB-231 and MDA-MB-468 cells.

5hmC

5hmC			NO vs. Control	
**Cell**	**Region**	**Gene**	**Δ β value**	**Δ Gene expression (logFC)**
MDA-MB-231	Promoter	GCNT1	1.0000	0.3159
		TNK1	0.2087	0.4851
		SLC1A7	0.3059	-2.4621
		NR2F1	0.1965	-0.4712
		SNORA22	0.0589	-1.0351
		NINJ2	0.1191	-1.0472
		GRIP1	1.0000	-0.5016
		PR11-83B20.1	0.0330	-1.4110
		RP1-153G14.4	-0.1299	1.6874
		SMG8	-1.0000	0.2850
		CTD-2521M24.9	-1.0000	0.3668
		ANKRD1	-0.1560	-0.4425
		NPTN-IT1	-0.3095	-0.4602
	Gene body	AC020571.3	0.2857	2.6076
		CSF3	0.1898	1.9440
		HSD11B1	0.2808	1.1932
		CTB-30L5.1	0.0934	2.7692
		UCA1	-0.3869	1.3948
		EGFR-AS1	-0.1474	1.2581
		AOC3	-0.0959	1.0845
		CTD-2003C8.2	-0.2986	1.0942
		RP1-153G14.4	-0.1299	1.6874
		RN7SL381P	-0.1806	-1.8645
	T. E.	TNXB	0.0932	1.6462
		LTF	-0.1000	-1.0578
	S.E.	-	-	-
MDA-MB-468	Promoter	COL5A2	0.0876	0.9874
		RP11-504A18.1	0.1474	-0.8253
		AC017006.2	0.2576	-2.9397
		PKI55	0.0015	-0.9377
		PCDHGB1	1.0000	-0.4591
		POLQ	-0.1982	0.2739
		PROM1	-0.0990	-0.6689
		CTB-147C13.1	-0.0883	-1.2587
	Gene body	GNG11	0.0611	1.2191
		RP11-61F12.1	0.0748	1.1646
		AC017006.2	0.1080	-2.9397
		RP3-332B22.1	-0.0528	2.4749
		RP11-327F22.2	-0.0503	1.2756
		IGSF10	-0.0884	-2.1161
	Typical enhancer	ADCYAP1	0.1446	1.9461
		ANKRD1	0.1795	1.1650
		DNER	0.1067	1.0611
		SOSTDC1	0.5841<br>0.0671	-1.8705
		MIR99AHG	0.2337	-1.1882
		ITGA9	0.1332	-1.0708
		FAM150B	0.1751	-1.1281
		CLIP4	-0.1246	1.0890
		TNFSF18	-0.2000	2.3731
		GLIS3	-0.2797	1.1439
		C8orf46	-0.1417	1.8990
	S.E.	LYPD5	0.0729	1.1175

Supplementary Table 3

Supplementary Tables 3: NO-regulated genes and their association with changes in hydroxymethylation (5hmC) at specific genomic loci. β-values (5hmC) at gene-regulatory loci (as determined by RnBeads) are paired with the expression changes in their associated genes in MDA-MB-231 and MDA-MB-468 cells.
