# Supplementary material for: Nitric oxide inhibits ten-eleven translocation DNA demethylases to regulate 5mC and 5hmC across the genome
Source: Nat Commun. 2025 Feb 18;16:1732. doi: 10.1038/s41467-025-56928-1 (PMC11836389; doi:10.1038/s41467-025-56928-1)
Supplement: Supplementary file 3 — Description of Additional Supplementary Files [file 41467_2025_56928_MOESM3_ESM.pdf]

Description of Additional Supplementary Files

File Name: Supplementary Data 1
Description: Data for Supplementary Figures 1. Modeling Simulations of NO, O2, and NO-donor concentrations
