# Supplementary material for: Nitric oxide inhibits ten-eleven translocation DNA demethylases to regulate 5mC and 5hmC across the genome
Source: Nat Commun. 2025 Feb 18;16:1732. doi: 10.1038/s41467-025-56928-1 (PMC11836389; doi:10.1038/s41467-025-56928-1)
Supplement: Supplementary file 5 — Reporting Summary [file 41467_2025_56928_MOESM5_ESM.pdf]

nature portfolio

Corresponding author(s): Douglas D. Thomas

Last updated by author(s): Dec 13, 2024

# Reporting Summary

Nature Portfolio wishes to improve the reproducibility of the work that we publish. This form provides structure for consistency and transparency in reporting. For further information on Nature Portfolio policies, see our Editorial Policies and the Editorial Policy Checklist.

## Statistics

For all statistical analyses, confirm that the following items are present in the figure legend, table legend, main text, or Methods section.

| n/a | Confirmed | |
|---|---|---|
| ☐ | ☒ | The exact sample size (*n*) for each experimental group/condition, given as a discrete number and unit of measurement |
| ☐ | ☒ | A statement on whether measurements were taken from distinct samples or whether the same sample was measured repeatedly |
| ☐ | ☒ | The statistical test(s) used AND whether they are one- or two-sided<br>*Only common tests should be described solely by name; describe more complex techniques in the Methods section.* |
| ☒ | ☐ | A description of all covariates tested |
| ☐ | ☒ | A description of any assumptions or corrections, such as tests of normality and adjustment for multiple comparisons |
| ☐ | ☒ | A full description of the statistical parameters including central tendency (e.g. means) or other basic estimates (e.g. regression coefficient) AND variation (e.g. standard deviation) or associated estimates of uncertainty (e.g. confidence intervals) |
| ☐ | ☒ | For null hypothesis testing, the test statistic (e.g. *F*, *t*, *r*) with confidence intervals, effect sizes, degrees of freedom and *P* value noted<br>*Give P values as exact values whenever suitable.* |
| ☒ | ☐ | For Bayesian analysis, information on the choice of priors and Markov chain Monte Carlo settings |
| ☒ | ☐ | For hierarchical and complex designs, identification of the appropriate level for tests and full reporting of outcomes |
| ☒ | ☐ | Estimates of effect sizes (e.g. Cohen's *d*, Pearson's *r*), indicating how they were calculated |

*Our web collection on statistics for biologists contains articles on many of the points above.*

## Software and code

Policy information about availability of computer code

| | |
|---|---|
| Data collection | Western Blot: FluorChem E system<br>Microplate Reader: BioTek Synergy HTX Multimode Reader<br>RNA Sequencing: NovaSeq 6000. Fastq files were generated and demultiplexed with the bcl2fastq v2.20 Conversion Software (Illumina).<br>oxRRBS: NovaSeq 6000. Fastq files were generated and demultiplexed with the bcl2fastq v2.20 Conversion Software (Illumina).<br>Density functional theory simulations and modeling: Gaussian 16 code92 to investigate the stability of nitrosyl complexes of Fe(II) active site models of TET2. As per the study of Lu et al.,41 theB97xD functional was employed as this Hamiltonian incorporates dispersion effects93, in conjunction with the def2-svp basis set94 for geometry optimizations and vibrational frequency calculation. For more accurate reaction energies, a single point calculation with a larger basis set – def2-tzvpp – was employed at the optimized stationary points. For the model complexes investigated all reasonable charge, spin and coordination states were examined. The results presented herein focus on the ground states identified for the proposed reaction intermediates. All complexes were fully optimized in the absence of any geometric or symmetry constraints. Quoted thermodynamic values assumed a temperature of 298.15 K and a pressure of 1 atm; enthalpic and entropic corrections used unscaled vibrational frequencies obtained at theB97xD/def2-svp level of theory. An SMD continuum solvation model (solvent = water) was employed for closer congruence with experimental conditions94.<br>The DNIC was modeled into PDB file 4NM642 using the modeling program COOT95. The model was energy minimized using REFMAC96 as implemented in CCP4i97. The NO bond length was restrained to 1.12 Å and the Fe-NO bond lengths restrained to 2.0Å.<br>Cell proliferation and invasion was measured using the Agilent xCELLigence Real-Time Cell Analysis system. |
| Data analysis | Microsoft Office 2019, ImageJ, R, and OriginPro were used to process data for publication.<br>RNA Sequencing: Read quality was assessed using MultiQC. Reads were aligned to hg38 GENCODE human genome (release 22) using STAR 2.5.2a82. Bam files were sorted using samtools83. Gene counts were generated using HTSeq84. Differential expression analysis was |

completed using edgeR v3.40.285. Gene Set Enrichment Analysis was completed using OmicPath (https://github.com/CBIIT-CGBB/OmicPath).
oxRRBS: Read quality was assessed using MultiQC86. Sequences were trimmed using TrimGalore (https://github.com/FelixKrueger/TrimGalore). Reads were aligned to hg38 using bowtie2 within Bismark (mapping efficiency ~62%) to count 5mC and 5hmC marks in a CpG context. Methylation analysis was performed using Bismark87 and Rnbeads88. Differential methylation analysis was performed using RnBeads, Dnmtools89, and MethylKit90. CpG annotation was performed with the annotatr package91 from BioConductor.

For manuscripts utilizing custom algorithms or software that are central to the research but not yet described in published literature, software must be made available to editors and reviewers. We strongly encourage code deposition in a community repository (e.g. GitHub). See the Nature Portfolio guidelines for submitting code & software for further information.

## Data

Policy information about availability of data

All manuscripts must include a data availability statement. This statement should provide the following information, where applicable:
- Accession codes, unique identifiers, or web links for publicly available datasets
- A description of any restrictions on data availability
- For clinical datasets or third party data, please ensure that the statement adheres to our policy

The authors declare that data supporting the findings of this study are available within the article, the accompanying source data files and the Supplementary Information. RNA-sequencing and RRBS-sequencing data are stored in the NCBI GEO repository under accession number GSE248151 which will become publicly available by 11/17/2027 or sooner with access instructions below. Source data are provided with this paper.
To review GEO accession GSE248151:
Go to https://www.ncbi.nlm.nih.gov/geo/query/acc.cgi?acc=GSE248151
Enter token uvmtssqqdzyhxmr into the box

## Research involving human participants, their data, or biological material

Policy information about studies with human participants or human data. See also policy information about sex, gender (identity/presentation), and sexual orientation and race, ethnicity and racism.

| | |
|---|---|
| Reporting on sex and gender | n.a. |
| Reporting on race, ethnicity, or other socially relevant groupings | n.a. |
| Population characteristics | n.a. |
| Recruitment | n.a. |
| Ethics oversight | n.a. |

Note that full information on the approval of the study protocol must also be provided in the manuscript.

# Field-specific reporting

Please select the one below that is the best fit for your research. If you are not sure, read the appropriate sections before making your selection.

☒ Life sciences ☐ Behavioural & social sciences ☐ Ecological, evolutionary & environmental sciences

For a reference copy of the document with all sections, see nature.com/documents/nr-reporting-summary-flat.pdf

# Life sciences study design

All studies must disclose on these points even when the disclosure is negative.

| | |
|---|---|
| Sample size | Sample size for each experiment is indicated in the figures or corresponding figure legends. No pre-determined sample size calculation were performed. Sample sizes were chosen based on standard protocol in the field. |
| Data exclusions | No data were excluded from the analyses. |
| Replication | All biological replicates are obtained from biologically independent experiments. All attempts at replication were successful. The experiment number has been clearly stated in the figure legends. |
| Randomization | Both in vitro samples and mice were randomly allocated into sample groups. |
| Blinding | Blinding was not possible for these experiments due to the sample extraction process. Data were collected in parallel to minimize bias. |

# Reporting for specific materials, systems and methods

We require information from authors about some types of materials, experimental systems and methods used in many studies. Here, indicate whether each material, system or method listed is relevant to your study. If you are not sure if a list item applies to your research, read the appropriate section before selecting a response.

## Materials & experimental systems

| n/a | Involved in the study |
|---|---|
| ☐ | ☒ Antibodies |
| ☐ | ☒ Eukaryotic cell lines |
| ☒ | ☐ Palaeontology and archaeology |
| ☐ | ☒ Animals and other organisms |
| ☒ | ☐ Clinical data |
| ☒ | ☐ Dual use research of concern |
| ☒ | ☐ Plants |

## Methods

| n/a | Involved in the study |
|---|---|
| ☒ | ☐ ChIP-seq |
| ☒ | ☐ Flow cytometry |
| ☒ | ☐ MRI-based neuroimaging |

# Antibodies

Antibodies used

TET1 (Rabbit) GeneTex, GTX124207, 1:1000
TET2 (Mouse) Active Motif, 61390, clone 21F11, 1.5 µg/mL
TET3 (Mouse) Abiocode, M1092-3, clone 4D5, 1:1000
ALKBH2 (Rabbit) Abcam, ab154859, clone EPR6177(2), 1:4000
DNMT1 (Rabbit) CellSignaling, 5032S, clone D63A6, 1:1000
DNMT3a (Rabbit) ABclonal, A19659, clone ARC0138, 1:1000
DNMT3b (Rabbit) ABclonal, A2899, 1:1000
NOS2 (Rabbit) ABclonal, A0312, 1:1000
β-Actin (Rabbit) CellSignaling, 4970S, clone 13E5, 1:1000
Anti-Mouse Secondary (Horse) CellSignaling, 7076S, 1:2000
Anti-Rabbit Secondary (Goat) CellSignaling, 7074S, 1:2000

Validation

TET1: manufacturer validated by knockdown with shRNA, orthogonal validation, and increased TET1 expression seen in TET1-transfected 293T cells
TET2: manufacturer validated, validated by knockdown with shRNA in PMID 27852070
TET3: manufacturer tested in human Jurkat
ALKBH2: manufacturer validated in several cell lines and tissues
DNMT1: manufacturer tested in 293 and F9 cells, knockdown by shRNA observed in PMID 37523636, knockdown by siRNA observed in PMID 28112215
DNMT3a: manufacturer tested and validated by knockout
DNMT3b: manufacturer tested in HCT116 cells
NOS2: manufacturer verified in RAW264.7 cells treated with LPS, validated in this manuscript in NOS2-overexpressing cells
β-Actin: manufacturer verified in various cell lines, highly cited (5964)
All antibodies were tested in our lab to recognize the expected molecular weight band.

# Eukaryotic cell lines

Policy information about cell lines and Sex and Gender in Research

Cell line source(s)

MDA-MB-231, MDA-MB-468, and PC-3 cell lines were obtained from ATCC. Primary cell PDX models (BCM-5998, BCM-3107, and BCM-3807) are each derived from female TNBC patients. U-251 cells were kindly provided by Dr. Bakhos A. Tannous (Dana Farber/Harvard Cancer Center). MDA-MB-231-VC/NOS2 cells were kindly provided by Dr. Sharon Glynn (University of Galway).

Authentication

Cell line authentication was performed using short tandem repeat analysis for MDA-MB-231 and MDA-MB-468 cell lines before sequencing.

Mycoplasma contamination

Cell lines tested negative for mycoplasma contamination.

Commonly misidentified lines
(See ICLAC register)

No commonly misidentified lines were used.

# Animals and other research organisms

Policy information about studies involving animals; ARRIVE guidelines recommended for reporting animal research, and Sex and Gender in Research

Laboratory animals

For cell-line derived studies, eight-week-old female athymic nude mice were supplied from the Frederick Cancer Research and Development Center Animal Production Area (Frederick, MD). For patient-derived xenograft studies, SCID Beige mice (Envigo) were used.dfds

| | |
|---|---|
| Wild animals | No wild animals were used. |
| Reporting on sex | Findings apply to only the female sex. This was deemed appropriate as TNBC has a significantly higher incidence in human females as compared to human males. |
| Field-collected samples | No field-collected samples were used. |
| Ethics oversight | Approved by the Committee on the Ethics of Animal Experiments of the National Cancer Institute, as well as Houston Methodist Hospital Research Institute Animal Care and Use Review Office. Protocols were performed in accordance with principles outlined in the Guide for the Care and Use of Laboratory Animals (Institute of Laboratory Animal Resources, National Research Council).The Institutional Animal Care and Use Committee (IACUC) approved the Animal Study Protocol. |

Note that full information on the approval of the study protocol must also be provided in the manuscript.

## Plants

| | |
|---|---|
| Seed stocks | n.a. |
| Novel plant genotypes | n.a. |
| Authentication | n.a. |
